# Reliability, Construct Validity, Acceptability and Feasibility of the BruxScreen

**DOI:** 10.1111/joor.70093

**Published:** 2025-11-05

**Authors:** Laurence J. Kessler, Merel C. Verhoeff, Tess Chin, Naichuan Su, Augustine Osman, Rahma Mungia, Frank Lobbezoo

**Affiliations:** ^1^ Department of Orofacial Pain and Dysfunction, Academic Centre for Dentistry Amsterdam (ACTA) University of Amsterdam and Vrije Universiteit Amsterdam Amsterdam the Netherlands; ^2^ Department of Oral Public Health, Academic Centre for Dentistry Amsterdam (ACTA) University of Amsterdam and Vrije Universiteit Amsterdam Amsterdam the Netherlands; ^3^ Department of Psychology The University of Texas at San Antonio San Antonio Texas USA; ^4^ Department of Periodontics, School of Dentistry The University of Texas Health Science at San Antonio San Antonio Texas USA; ^5^ Department of Orofacial Pain and Jaw Function, Faculty of Odontology Malmö University Malmö Sweden

**Keywords:** bruxism, data accuracy, mass screening, reproducibility of results, self report, surveys and questionnaires

## Abstract

**Background:**

The recently developed BruxScreen consists of two parts: BruxScreen‐Q (self‐report questionnaire) and BruxScreen‐C (clinical assessment).

**Objectives:**

To test the intra‐ and inter‐rater reliability, construct validity, acceptability and feasibility of the BruxScreen‐Q and BruxScreen‐C and assess their concordance among Dutch dental students.

**Methods:**

88 out of 109 potentially eligible dental master students completed a set of questionnaires two times (Q1; Q2) and participated in two clinical workshops (CE1; CE2), using the BruxScreen‐Q and BruxScreen‐C, respectively. Intra‐rater reliability of the BruxScreen‐Q and concordance between the BruxScreen‐Q and BruxScreen‐C were assessed using Cohen's (weighted) Kappa. Intra‐ and inter‐rater reliability of the BruxScreen‐C were analysed using intraclass correlation coefficients calculated from generalised linear mixed‐effects models. Construct validity of the BruxScreen‐Q was tested using Spearman's Rank Correlation or Mann–Whitney *U* test based on hypothesis testing. Acceptability and feasibility of the BruxScreen were assessed using descriptive statistics.

**Results:**

Intra‐rater reliability for BruxScreen‐Q was fair to substantial. Intra‐ and inter‐rater reliability for BruxScreen‐C varied from poor to excellent. BruxScreen‐Q showed moderate construct validity, based on the acceptable consistency between the actual and hypothesised effect size of the questionnaire items. BruxScreen‐Q (Q2) and BruxScreen‐C (CE2) were found both acceptable and feasible by a majority of the students. There was no agreement between subject‐based bruxism according to the BruxScreen‐Q and clinically based bruxism according to the BruxScreen‐C.

**Conclusion:**

The BruxScreen demonstrates acceptable reliability, construct validity, acceptability and feasibility in assessing both subject‐based bruxism and clinically based bruxism. However, there is a discrepancy between self‐reported bruxism and the clinicians' diagnosis.

## Graphical abstract

This study aimed to evaluate the reliability, construct validity, acceptability and feasibility of the BruxScreen‐Q and the BruxScreen‐C, as well as their concordance in a cohort of Dutch dental students.

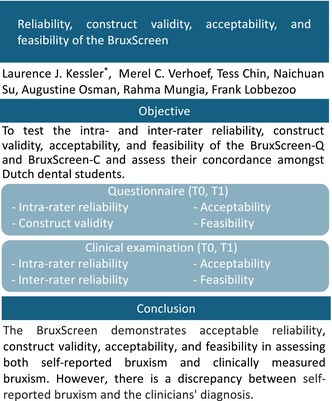

## 1 Background

Bruxism is defined as ‘a repetitive jaw‐muscle activity characterised by clenching or grinding of the teeth and/or by bracing or thrusting of the mandible’ [1]. Bruxism can occur during wakefulness (awake bruxism [AB]), and while sleeping (sleep bruxism [SB]) [1, 2]. The reported prevalence varies, with a study in the Dutch adult population estimating a prevalence of 5.0% for AB and 16.5% for SB [3]. Bruxism can be a motor behaviour without pathological consequences. For some people, however, it can be a risk factor for certain clinical consequences [4]. For example, bruxism has been associated with tooth wear, orofacial pain and failure of dental restorations [1, 2].

The assessment of bruxism is complex, involving self‐reported questionnaires, clinical evaluations and device‐based measurements such as electromyographic recordings or polysomnography [2]. Self‐reports allow for the identification of subject‐based bruxism, while clinical examination can lead to a clinically based diagnosis. However, a device‐based diagnosis requires instrumental assessment—electromyographic recordings for AB and polysomnography for SB [5]. Despite these classification criteria, challenges remain in establishing valid cut‐off points for clinically significant AB and SB [6]. This has led to calls for a standardised, clinically practical assessment tool that balances accuracy with feasibility in routine dental settings.

In response to this need, the Standardised Tool for the Assessment of Bruxism (STAB) was introduced in 2024 [7, 8]. However, due to its comprehensiveness, the STAB does not fully comply with the A4 principle (viz., Accurate, Applicable, Affordable and Accessible) [9]. To address this limitation, a shorter and more practical screening tool, the BruxScreen, was developed. Unlike the STAB, the BruxScreen is applicable in general dental practices to detect signs or negative consequences of bruxism. The BruxScreen consists of two parts: (1) the BruxScreen‐Q, involving self‐report questionnaires, and (2) the BruxScreen‐C, which concerns a clinical assessment. The BruxScreen‐C was pilot‐tested at two university clinics, and face validity was established [9]. However, before widespread adoption, further testing is needed to establish its reliability, construct validity, acceptability and feasibility for the BruxScreen‐Q and the BruxScreen‐C.

This study's aims are to test: (1) the reliability of the BruxScreen‐Q and BruxScreen‐C, (2) the construct validity of the BruxScreen‐Q, (3) the acceptability and feasibility of the BruxScreen‐Q and BruxScreen‐C, and (4) the concordance between the BruxScreen‐Q and BruxScreen‐C. Given that the BruxScreen was developed following the A4 principle and incorporates validated assessment components, it is hypothesised that the BruxScreen‐Q and BruxScreen‐C will demonstrate strong reliability, construct validity, acceptability and feasibility [9]. Furthermore, based on prior findings indicating a positive correlation between self‐reported bruxism and clinical diagnoses of awake bruxism (clenching type) [10], it is expected that the BruxScreen‐Q and BruxScreen‐C will show positive concordance. If confirmed, this would provide a significant advancement in the field by offering a practical, standardised and evidence‐based tool for early bruxism detection and management in dental settings.

## 2 Methods

### 2.1 Study Design and IRB Approval

The BruxScreen validation project is an observational study. The Institutional Review Board (IRB) of the Academic Centre for Dentistry Amsterdam (ACTA) approved the ‘BruxScreen validation project’ (Protocol number: 2023‐49109).

### 2.2 Study Sample

Out of 109 potentially eligible first‐year dental master students of ACTA, who participated in a course on orofacial pain and dysfunction during the academic year 2023–2024, only those with complete data sets and giving their informed consent were included. This sample was selected to take advantage of a controlled educational setting, ensuring that all participants had a consistent level of training and exposure to bruxism assessment.

### 2.3 Procedure

**FIGURE 1:**
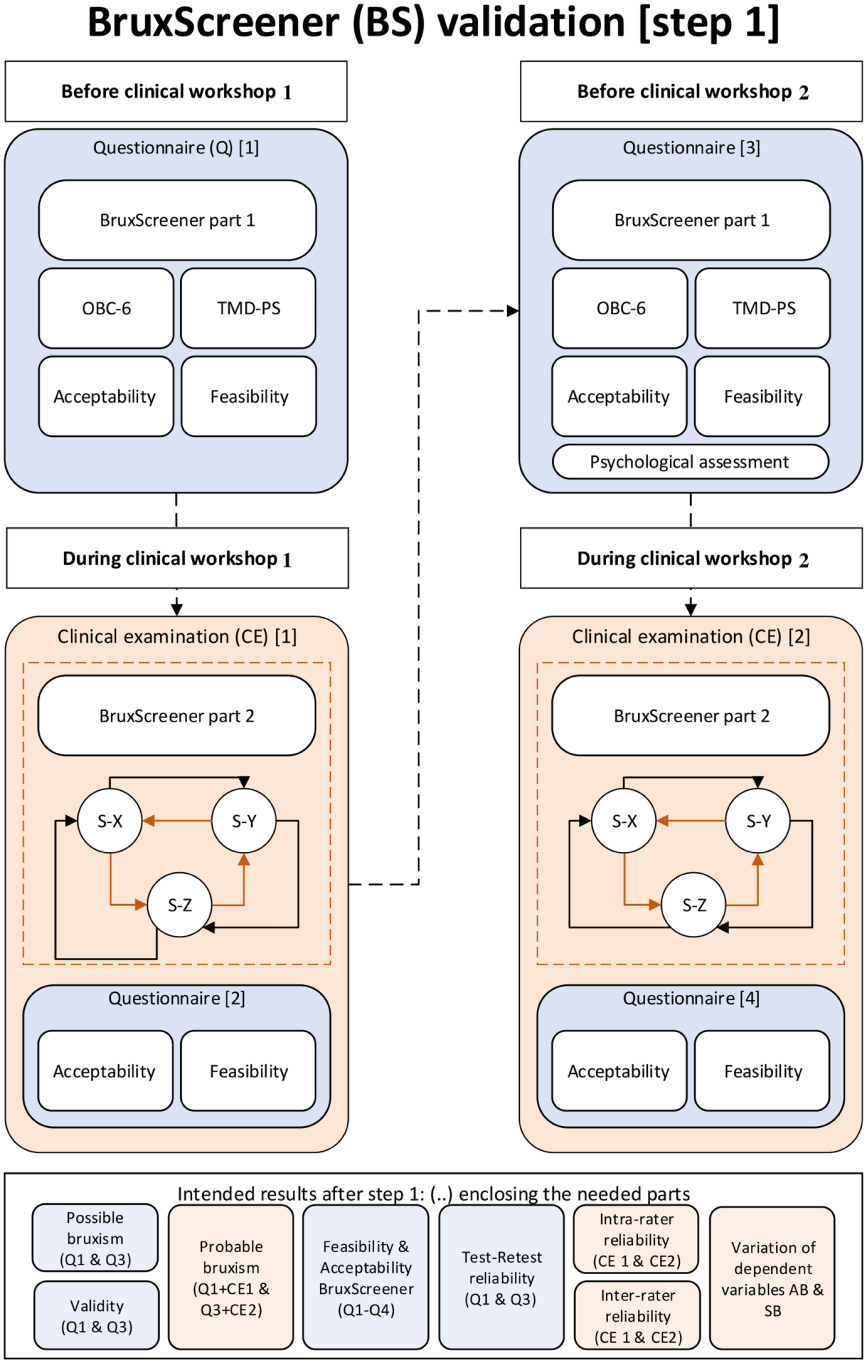
Flowchart for the BruxScreen workshops. Before Clinical workshop 1, the participating students filled in the BruxScreen‐Q, the TMD Pain Screener (TMD‐ps), the Oral Behaviour Checklist 6 (OBC‐6), and a custom‐made questionnaire for the acceptability and feasibility of the BruxScreen‐Q. During both clinical workshops, the students were coupled in groups of three (S‐X, S‐Y and S‐Z). One of the three students examined another student, while the third student waited outside the examination room. After that, students alternated roles, ensuring each student fulfilled each role twice with a different student. The student in the role of the dentist filled in the BruxScreen‐C, and a custom‐made questionnaire for the acceptability and feasibility of the BruxScreen‐C.

The study was conducted during two clinical workshops (Clinical workshop 1 and 2) (Figure 1), separated by a 2‐week interval. Prior to the clinical workshops, each student completed a set of four online questionnaires: (1) the Temporomandibular Disorders Pain Screener (TMD‐PS) [11], (2) the Oral Behaviour Checklist 6 (OBC‐6) [12], (3) the BruxScreen‐Q [9], and (4) a custom‐made questionnaire for the acceptability and feasibility of the BruxScreen‐Q (see Appendix A). To maintain data integrity, each student was assigned a randomised participant code, ensuring anonymity and preventing traceability of the collected data. After completing the questionnaires, students attended the clinical workshops. During Clinical workshop 1, the students were coupled in groups of three, rotating between the roles of dentist and patient. In the dentist role, each student performed a clinical assessment using the BruxScreen‐C [9], and in addition, completed a custom‐made questionnaire for the acceptability and feasibility of the BruxScreen‐C (see Appendix A). After that, they rotated roles. This process was repeated 2 weeks later (Clinical workshop 2) to measure the intra‐rater reliability. Eventually, the following data were available from each student: two times the completed sets of questionnaires, including the BruxScreen‐Q, and four times the completed sets of questionnaires filled in during the clinical workshops, including the BruxScreen‐C. All data were securely collected using Castor Electronic Data Capture (EDC) software (Castor EDC, New York, USA), ensuring a standardised, efficient and error‐free data collection process.

During Clinical workshop 1, students only received logistical guidance and no explanation about the content of the BruxScreen‐C, while at the conclusion of Clinical workshop 2, explanations were offered by the teacher. Moreover, the successive students in the role of the dentist were not allowed to compare or discuss their patient findings. Additionally, there were 2 weeks between Clinical workshop 1 and Clinical workshop 2, so the students in the role of the dentist would not remember the details of their previous patients' findings. Lastly, the student in the role of the patient was not allowed to interfere with the examination of the student in the role of the dentist.

### 2.4 Instruments

The BruxScreen consists of two parts: (1) the BruxScreen‐Q, which involves self‐report questionnaires, and (2) the BruxScreen‐C, which concerns a clinical assessment, to be performed by the dentist. To test the reliability of the BruxScreen‐Q and BruxScreen‐C, as well as the construct validity of the BruxScreen‐Q, several additional instruments were used. These included the TMD‐PS and the OBC‐6. Further, custom questionnaires were created to assess the acceptability and feasibility of both the BruxScreen‐Q and the BruxScreen‐C (see Appendix A). Detailed descriptions of the instruments are provided below.

#### 2.4.1 BruxScreen‐Q

The BruxScreen‐Q was designed to assess both bruxism behaviours and their most frequently observed negative consequences, such as signs and symptoms of temporomandibular disorders (TMDs) [9]. The BruxScreen‐Q comprises two questions. There are six response options available, ranging from ‘never’ (zero points) to ‘always’ (four points). In addition, the option ‘I don't know’ was available. To meet the cut‐off for subject‐based bruxism based on the BruxScreen‐Q, a minimum score of ‘often’ was needed at least once for questions 1a, 1b, 1c, 1d and/or 1e, on both occasions.

#### 2.4.2 BruxScreen‐C

With the BruxScreen‐C, the dentist evaluates potential extra‐oral and intra‐oral indicators that could be associated with bruxism, particularly focusing on masseter muscle hypertrophy, lesions or hyperkeratosis of the intra‐oral soft tissues, and tooth wear [9]. The first two questions of the extra‐oral and intra‐oral inspection have two response options: ‘absent’ (zero points) and ‘present’ (one point). For the third question, the Dutch Tooth Wear Screening Index (DTWSI) is measured. The highest score of tooth wear is noted per sextant: ‘no wear’ (zero points); ‘visible wear within the enamel’ (one point); ‘visible wear with dentin exposure and loss of clinical crown height of ≤ 1/3’ (two points); ‘loss of crown height > 1/3 but ≤ 2/3’ (three points); and ‘loss of clinical crown height ≥ 2/3’ (four points) [13]. The following cut‐off criterion was used to assess clinically based bruxism based on the BruxScreen‐C: ‘≥ 1× “present” at question 2a, b and/or c AND DTWSI ≥ 1’.

#### 2.4.3 Temporomandibular Disorders Pain Screener (TMD‐PS)

The TMD‐PS comprises three TMD‐related questions [11]. The initial question of the TMD‐PS has three response options that are measured on an ordinal scale: ‘no pain’ (zero points); ‘pain comes and goes’ (one point); and ‘pain is always present’ (two points). The second question has two response options: ‘no’ (zero points) and ‘yes’ (one point). As for the third question of the TMD‐PS, there are three response options available: ‘pain unchanged’ (zero points); ‘yes, pain reduced’ (one point); and ‘yes, pain increased’ (two points), applicable to each of the four subquestions. For the purpose of our data analysis, the categories of the first two questions were dichotomised into two groups: absence of pain and presence of pain (which includes both intermittent and chronic pain). The categories of the third question were re‐coded as follows: ‘yes, pain reduced’ (zero points); ‘pain unchanged’ (one point); and ‘yes, pain increased’ (two points).

#### 2.4.4 Oral Behaviour Checklist 6 (OBC‐6)

The OBC‐6 comprises two questions regarding bruxism activities while sleeping and four questions regarding bruxism activities while awake. The questions assess the frequency of engaging in these activities [12]. The questions regarding bruxism activities while sleeping are scored from ‘never’ (zero points) to ‘four to seven nights per week’ (four points). The questions regarding bruxism activities while awake are scored from ‘never’ (zero points) to ‘always’ (four points).

#### 2.4.5 Acceptability and Feasibility of the BruxScreen‐Q and BruxScreen‐C

The custom‐made questionnaires for the acceptability and feasibility of the BruxScreen‐Q and BruxScreen‐C comprise three and four questions regarding the acceptability, respectively. Furthermore, there are two questions regarding the feasibility for both the BruxScreen‐Q and the BruxScreen‐C. The questions were scored using a Likert scale, ranging from ‘strongly disagree’ (zero points) to ‘strongly agree’ (four points). The cut‐off values for both the acceptability and the feasibility of the BruxScreen‐Q were set at nine and six points; for the BruxScreen‐C, they were set at twelve and six points, respectively; this corresponds to a minimum response of ‘agree’ with each question.

### 2.5 Statistical Analysis

To test the reliability, construct validity, acceptability and feasibility of the BruxScreen‐Q and BruxScreen‐C, descriptive statistics (e.g., mode, median and mean) were calculated first. Below, more detailed information about the subsequent statistical analyses is provided.

#### 2.5.1 Reliability

To analyse the reliability, a distinction was made between intra‐rater reliability (viz., the consistency of data measured on two different points in time by one rater [14]) and inter‐rater reliability (viz., the consistency of data measured by two raters [14]). Moreover, as the BruxScreen is developed as a screening tool, the intra‐ and inter‐rater reliability of the overall conclusion based on the questions in the tool (viz., subject‐based and clinically based bruxism) was analysed accordingly. To assess intra‐rater reliability and inter‐rater reliability of the BruxScreen‐C, intraclass correlation coefficients (ICCs) were calculated using generalised linear mixed‐effects models (GLMMs). The ICCs were calculated based on the estimated variance components of the models. For the BruxScreen‐Q, Cohen's Kappa was used if the questions were dichotomised. For ordinal questions, a weighted Cohen's Kappa was used. The specifics are described in the following sections.

BruxScreen‐Q: To measure the intra‐rater reliability, weighted Cohen's Kappa was used. The response ‘I don't know’ was removed from the analysis. The intra‐rater reliability for diagnosing the subject‐based bruxism was measured using Cohen's Kappa.

The outcomes of the inter‐ and intra‐rater reliability were qualified as follows, based on the kappa values: no agreement (< 0); none to slight agreement (0.01–0.2); fair agreement (0.21–0.4); moderate agreement (0.41–0.6); substantial agreement (0.61–0.8); and almost perfect agreement (0.81–1) [15].

BruxScreen‐C: For intra‐rater reliability, GLMMs were fitted including both rater ID and rater‐by‐timepoint interaction as random effects, which allows to capture the consistency of each rater's scores across different timepoints, while accounting for between‐rater variability. For inter‐rater reliability, models were fitted including rater ID as a random effect and adjusted for the timepoint (i.e., Clinical workshop 1 and Clinical workshop 2) as a fixed effect to account for systematic temporal variation.

The outcomes of the inter‐ and intra‐rater reliability were qualified as follows, based on the ICC values: poor reliability (< 0.5), moderate reliability (0.50–0.75), good reliability (0.75–0.9) and excellent reliability (> 0.90) [16].

#### 2.5.2 Construct Validity

To test the construct validity (viz., the level of consistency of the scores of an instrument with the hypotheses, based on the premise that the instrument accurately measures what it is intended to measure [17]), the hypothesis testing approach was used. To that end, hypotheses were formulated for the BruxScreen‐Q based on the two validated questionnaires (viz., OBC‐6 and TMD‐PS) (Appendix B—Table B1). The hypotheses were based on our clinical and research knowledge and experience. The OBC‐6 was used to validate the questions of the BruxScreen‐Q regarding bruxism, while the TMD‐PS was used to validate the TMD‐related questions of the BruxScreen‐Q. Each hypothesis was analysed using either Spearman's Rank Correlation or the Mann–Whitney *U* test, depending on the outcome measures. Spearman's Rank Correlation was used when both outcome measures were ordinal, while the Mann–Whitney *U* test was used when one outcome measure was dichotomous and the other was ordinal. Data from Clinical workshop 2 was used to assess the construct validity, as students likely acquired enhanced knowledge about bruxism and TMD pain during the course on orofacial pain and dysfunction.

The correlation coefficient of Spearman's Rank Correlation analysis is regarded as the effect size of the analysis. To interpret the Spearman's Rank Correlation coefficient, the scale of Evans [18] was used: very weak (0.00–0.19), weak (0.20–0.39), moderate (0.40–0.59), strong (0.60–0.79) and very strong (0.80–1.0). To interpret the effect size of the Mann–Whitney *U* test, the following criteria were used [19]: small effect (effect size *r* < 0.30), medium effect (effect size *r* = 0.30–0.50) and large effect (effect size *r* > 0.50).

#### 2.5.3 Acceptability and Feasibility

The mode, median and interquartile range (IQR) were calculated for each question of the custom‐made questionnaires for the acceptability and feasibility of the BruxScreen‐Q and BruxScreen‐C. Furthermore, the mean durations for reading the explanations and completing the BruxScreen‐Q and BruxScreen‐C were calculated. Additionally, the mean duration for completing the BruxScreen‐Q, TMD‐PS and OBC‐6 questionnaires was also assessed.

BruxScreen‐Q: The custom‐made questionnaires for the acceptability and feasibility of the BruxScreen‐Q were completed twice. The percentage of people who met the cut‐off criterion for the acceptability and feasibility of the BruxScreen‐Q was measured for Clinical workshop 1 and Clinical workshop 2.

BruxScreen‐C: The custom‐made questionnaires for the acceptability and feasibility of the BruxScreen‐C were completed two times, i.e., during Clinical workshop 1 and Clinical workshop 2, by the student in the role of the dentist. For each workshop, the data of the custom‐made questionnaires for the acceptability and feasibility were pooled, respectively. The percentage of people who met the cut‐off criterion for the acceptability and feasibility of the BruxScreen‐C was measured for Clinical workshop 1 and Clinical workshop 2.

#### 2.5.4 Concordance Between the BruxScreen‐Q and BruxScreen‐C

To assess concordance between the BruxScreen‐Q and the BruxScreen‐C, Cohen's Kappa was used to measure agreement between the two tools, ensuring that findings from self‐reported questionnaires were consistent with clinical evaluations. Only complete datasets, containing both BruxScreen‐Q and BruxScreen‐C assessments from both workshops (Clinical workshop 1 and Clinical workshop 2), were included in the concordance analysis. If either one of them was missing, the total set was excluded from this analysis.

Statistical analysis was done using IBM SPSS Statistics 29.0 software (IBM Corp., Armonk, New York, USA).

## 3 Results

### 3.1 Demographics

In total, 109 potentially eligible first‐year dental master students of ACTA (year 4 of 6 study years), participated in the course on orofacial pain and dysfunction during the academic year 2023–2024. Two students did not give their informed consent and were therefore excluded. Further, only those with complete datasets were included. Thus, the analysis of the BruxScreen‐Q included 88 participants, of whom 24 were male, and one participant identified as a gender other than male or female. The age ranged between 20 and 31 years (mean ± SD = 22.9 ± 2.4 years). For the BruxScreen‐C, 62 participants were included, of which 18 were male, and two participants did not fill in their gender. Their age ranged between 20 and 30 years (mean ± SD = 22.8 ± 2.3 years). To analyse the concordance between the BruxScreen‐Q and BruxScreen‐C, 54 participants were included.

Based on self‐report, 30.7% of the students (*N* = 27) had subject‐based bruxism. Among these, 18.2% (*N* = 16) reported AB, while 23.9% (*N* = 21) experienced SB. Based on the clinical assessment, 35.2% of the students (*N* = 22) had clinically based bruxism. Only 5.6% of the students (*N* = 3) presented themselves with bruxism based on both self‐report and clinical examination (Table 1). Additionally, there was no agreement between the outcome of subject‐based bruxism according to the BruxScreen‐Q and clinically based bruxism according to the BruxScreen‐C (kappa value = −0.138; 95% CI = −0.38 to 0.10; *p* = 0.294).

**TABLE 1:** Demographics and prevalence of bruxism of dental master students of the Academic Centre for Dentistry Amsterdam.

Questionnaire	*n* = 88	Gender (*n* [%])	Male	24 (27.3%)
			Female	63 (71.6%)
			Other	1 (1.1%)
		Age (M ± SD)		22.9 ± 2.4
		Subject‐based bruxism (*n* [%])		27 (30.7%)
		Sleep bruxism (*n* [%])		21 (23.9%)
		Awake bruxism (*n* [%])		16 (18.2%)
Clinical	*n* = 62	Gender (*n* [%])^a^	Male	18 (29.0%)
			Female	42 (67.7%)
		Age (M ± SD)^b^		22.8 ± 2.3
		Clinically based bruxism (*n* [%])		22 (35.5%)
Both	*n* = 54	Concordance Q + C (*n* [%])		3 (5.6%)

Abbreviations: Clinically based bruxism, clinical examination; m, mean age in years; *n*, number of participants; N/A, not applicable; SD, standard deviation; Subject‐based bruxism, self‐report.

^a^ Two participants did not fill in their gender.

^b^ Two participants did not fill in their age.

### 3.2 Reliability

The average intra‐rater and inter‐rater reliability of each question of the BruxScreen‐Q and BruxScreen‐C are presented below.

BruxScreen‐Q: For the intra‐rater reliability of the BruxScreen‐Q, eight of the 31 questions demonstrated a substantial level of agreement. The majority of the questions, i.e., 22 out of 31, showed a moderate level of agreement. Furthermore, one out of 31 questions showed a fair level of agreement. The level of agreement for the outcome of ‘subject‐based bruxism’ was moderate (Table 2).

**TABLE 2:** Intra‐rater reliability of the BruxScreen‐Q.

Intra‐rater reliability				
*n* = 88	Level of agreement	*K* value	95% CI	*p*
1a. How often do you clench your teeth, with or without contact between upper and lower teeth, during sleep?	Substantial	0.65	0.53–0.76	< 0.001
1b. How often do you grind your teeth during sleep?	Substantial	0.77	0.67–0.87	< 0.001
1c. How often do you clench your teeth, with contact between upper and lower teeth, while awake?	Substantial	0.70	0.59–0.81	< 0.001
1d. How often do you clench your teeth, without contact between upper and lower teeth, while awake?	Substantial	0.61	0.46–0.76	< 0.001
1e. How often do you grind your teeth while awake?	Moderate	0.58	0.38–0.79	< 0.001
2a1. How often do you experience pain in your temple, face, jaw or jaw point upon awaking?	Substantial	0.63	0.47–0.78	< 0.001
2a2. How often do you experience unpleasantness in your temple, face, jaw or jaw point upon awaking?	Substantial	0.64	0.50–0.77	< 0.001
2a3. How often do you experience sensitivity in your temple, face, jaw or jaw point upon awaking?	Moderate	0.58	0.44–0.73	< 0.001
2a4. How often do you experience tiredness in your temple, face, jaw or jaw point upon awaking?	Moderate	0.60	0.46–0.73	< 0.001
2a5. How often do you experience tension in your temple, face, jaw or jaw point upon awaking?	Moderate	0.56	0.42–0.70	< 0.001
2a6. How often do you experience stiffness in your temple, face, jaw or jaw point upon awaking?	Moderate	0.46	0.33–0.60	< 0.001
2a7. How often do you experience pain in your temple, face, jaw or jaw point upon at any other time?	Moderate	0.53	0.34–0.72	< 0.001
2a8. How often do you experience unpleasantness in your temple, face, jaw or jaw point upon at any other time?	Moderate	0.50	0.34–0.65	< 0.001
2a9. How often do you experience sensitivity in your temple, face, jaw or jaw point upon at any other time?	Moderate	0.55	0.39–0.71	< 0.001
2a10. How often do you experience tiredness in your temple, face, jaw or jaw point upon at any other time?	Moderate	0.58	0.47–0.69	< 0.001
2a11. How often do you experience tension in your temple, face, jaw or jaw point upon at any other time?	Moderate	0.60	0.46–0.74	< 0.001
2a12. How often do you experience stiffness in your temple, face, jaw or jaw point upon at any other time?	Moderate	0.50	0.36–0.65	< 0.001
2b1. How often do you experience pain in your temple, face, jaw or jaw joint when you open your mouth or chew during meals?	Moderate	0.52	0.31–0.72	< 0.001
2b2. How often do you experience unpleasantness in your temple, face, jaw or jaw joint when you open your mouth or chew during meals?	Moderate	0.57	0.38–0.76	< 0.001
2b3. How often do you experience sensitivity in your temple, face, jaw or jaw joint when you open your mouth or chew during meals?	Moderate	0.46	0.30–0.61	< 0.001
2b4. How often do you experience tiredness your temple, face, jaw or jaw joint when you open your mouth or chew during meals?	Fair	0.39	0.21–0.57	< 0.001
2b5. How often do you experience tension in your temple, face, jaw or jaw joint when you open your mouth or chew during meals?	Moderate	0.48	0.28–0.69	< 0.001
2b6. How often do you experience stiffness in your temple, face, jaw or jaw joint when you open your mouth or chew during meals?	Moderate	0.53	0.38–0.68	< 0.001
2b7. How often do you experience pain in your temple, face or jaw point when you open your mouth or chew at any other time?	Substantial	0.68	0.51–0.85	< 0.001
2b8. How often do you experience unpleasantness in your temple, face or jaw point when you open your mouth or chew at any other time?	Moderate	0.51	0.34–0.68	< 0.001
2b9. How often do you experience sensitivity in your temple, face or jaw point when you open your mouth or chew at any other time?	Moderate	0.53	0.31–0.74	< 0.001
2b10. How often do you experience tiredness in your temple, face or jaw point when you open your mouth or chew at any other time?	Moderate	0.42	0.25–0.59	< 0.001
2b11. How often do you experience tension in your temple, face or jaw point when you open your mouth or chew at any other time?	Moderate	0.41	0.23–0.59	< 0.001
2b12. How often do you experience stiffness in your temple, face or jaw point when you open your mouth or chew at any other time?	Moderate	0.51	0.31–0.70	< 0.001
2c1. How often does your jaw lock or become stuck during meals?	Moderate	0.56	0.14–0.97	< 0.001
2c2. How often does your jaw lock or become stuck at any other time?	Substantial	0.61	0.29–0.93	< 0.001
Subject‐based bruxism (Q)	Moderate	0.54	0.36–0.72	< 0.001

*Note:* Subject‐based bruxism (Q) based on BruxScreen‐Q with a cut‐off a minimum score of ‘often’ at least once for questions 1a, 1b, 1c, 1d and/or 1e, on both days.

Abbreviations: 95% CI, 95% confidence interval; *K*, Kappa value; *N*, number of participants.

BruxScreen‐C: For the intra‐rater reliability of the BruxScreen‐C, one of the 15 questions demonstrated an excellent reliability, four out of 15 questions showed a moderate reliability, and nine out of 15 questions had a poor reliability. The reliability for measuring the outcome ‘clinically based bruxism’ between Clinical workshop 1 and Clinical workshop 2, according to the BruxScreen‐C, was poor. Table 3 provides a detailed overview of the intra‐rater reliability and inter‐rater reliability outcomes of the BruxScreen‐C.

**TABLE 3:** Intra‐rater reliability and inter‐rater reliability of the BruxScreen‐C.

*N* = 62	Intra‐rater reliability		Inter‐rater reliability	
	Reliability	ICC	Reliability	ICC (overall adjusted by time)
1a. Masseter muscle hypertrophy (observed while the muscles are relaxed)	Poor	0.207	Excellent	0.940
1b. Masseter muscle hypertrophy (observed while the muscles are contracted)	Moderate	0.669	Moderate	0.679
2a. Lip (indentations)	Poor	0.312	Poor	0.343
2b. Cheek (linea alba)	Poor	0.422	Poor	0.426
2c. Tongue (indentations)	Poor	0.208	Poor	0.209
2d. Tongue (traumatic lesions)		—^a^		—^a^
2e. Alveolar bone (exostoses/tori)	Excellent	0.935	Excellent	0.935
3c. The observed tooth wear	Poor	0.478	Poor	0.488
DTWSI: Sextant 1	Poor	0.495	Moderate	0.509
DTWSI: Sextant 2i	Moderate	0.527	Moderate	0.531
DTWSI: Sextant 2p	Moderate	0.559	Moderate	0.576
DTWSI: Sextant 3	Moderate	0.524	Moderate	0.534
DTWSI: Sextant 4	Poor	0.393	Poor	0.396
DTWSI: Sextant 5	Poor	0.391	Poor	0.400
DTWSI: Sextant 6	Poor	0.326	Poor	0.330
Clinically based bruxism (C)	Poor	0.480	Poor	0.484

*Note:* Clinically based bruxism (C) based on BruxScreen‐C with the cut‐off ‘≥ 1× present (2a, b and/or c) AND DTWSI ≥ 1’.

Abbreviations: ICC, Intraclass correlation coefficient; *N*, number of participants.

^a^ An ICC value could not be generated via GLMM for either intra‐rater or inter‐rater reliability because all cases were scored as ‘No’ in Clinical workshop 1.

For the inter‐rater reliability of the BruxScreen‐C, two of the 15 questions showed an excellent reliability. Five out of 15 questions had a moderate reliability, while for another seven out of 15 questions, the reliability was poor. Additionally, the reliability for measuring the outcome ‘clincally based bruxism’ between the two raters, according to the BruxScreen‐C, was poor (Table 3).

### 3.3 Construct Validity

The validity analysis between the BruxScreen‐Q and the OBC‐6 showed that two out of five items had a higher effect size than the hypothesised effect size. Additionally, one item showed an effect size that equals the hypothesised effect size. Lastly, for two out of five items, the actual effect size was lower than the hypothesised effect size (Table 4).

**TABLE 4:** Validity analysis of the BruxScreen‐Q with the OBC‐6 and TMD‐PS.

BruxScreen‐Q	Comparable validated question		*N*	Level of correlation (*ρ*)	Effect size (*r*)	Hypothesised level of correlation	Hypothesised effect size
1a. How often do you clench your teeth during sleep?	OBC‐6	1. How often do you clench or grind teeth when asleep, based on any information you may have?	75	0.79 (strong)	N/A	0.4 (moderate)	N/A
1b. How often do you grind your teeth during sleep?	OBC‐6	1. How often do you clench or grind teeth when asleep, based on any information you may have?	80	0.56 (moderate)	N/A	0.4 (moderate)	N/A
1c. How often do you clench your teeth while awake?	OBC‐6	4. How often do you clench teeth together during waking hours?	87	0.84 (very strong)	N/A	0.6 (strong)	N/A
1d. How often do you grind your teeth while awake?	OBC‐6	3. How often do you grind your teeth during waking hours?	83	0.45 (moderate)	N/A	0.6 (strong)	N/A
1e. How often do you light press, touch or hold teeth together while awake other than while eating (i.e., contact between upper and lower teeth)?	OBC‐6	5. How often do you press, touch or hold teeth together other than while eating (i.e., contact between upper and lower teeth)	87	0.26 (weak)	N/A	0.6 (strong)	N/A
2a1. How often do you experience pain in your temple, face or jaw point upon awaking?	TMD‐PS	2. In the last 30 days, have you had pain or stiffness in your jaw on awakening?	88	N/A	0.43 (medium)	N/A	0.3 (medium)
2a7. How often do you experience pain in your temple, face or jaw point upon awaking at any other time?	TMD‐PS	1. In the last 30 days, which of the following describes any pain in your jaw or temple area on either side? (No pain, pain comes and goes, pain is always present)	66	N/A	0.65 (large)	N/A	0.5 (large)
2b1. How often do you experience pain in your temple, face or jaw point when you open your mouth or chew? (Answers meals related)	TMD‐PS	3a. In the last 30 days, did chewing hard or tough food change any pain (i.e., make it better or make it worse) in your jaw or temple area on either side?	88	0.56 (moderate)	N/A	0.4 (moderate)	N/A
2b7. How often do you experience pain in your temple, face or jaw point when you open your mouth or chew? (Answers related to opening the mouth)	TMD‐PS	3b. In the last 30 days, did opening your mouth or moving your jaw forward or to the side change any pain (i.e., make it better or make it worse) in your jaw or temple area on either side?	88	0.361 (weak)	N/A	0.4 (moderate)	N/A

*Note:* The construct validity was determined through Spearman's rank correlation (*ρ* = Spearman's rank correlation coefficient) for questions 1a, 1b, 1, 1d, 1e, 2b1 and 2b7. For questions 2a1 and 2a7, the Mann–Whitney *U* test (*r* = effect size) was performed.

Abbreviations: *N*, number of participants; N/A, not applicable; OBC‐6, Oral Behaviour Checklist 6; TMD‐PS, Temporomandibular Disorders Pain Screener.

In the validity analysis comparing the BruxScreen‐Q and the TMD‐PS, the effect size for three out of four items was equal to the hypothesised effect size. However, for one item, a lower effect size was found than the hypothesised effect size (Table 4).

### 3.4 Acceptability and Feasibility

The mode and median for the acceptability and feasibility questions were all 3 (viz., ‘agree’) on a 5‐point Likert scale. According to the cut‐off, this can be interpreted as an acceptable outcome for the acceptability and feasibility of the BruxScreen. The accompanying interquartile ranges per question are given in Table 5.

**TABLE 5:** Acceptability and feasibility BruxScreen.

	*N*			Mean (%)	Mode	Median	IQR
Acceptability	88	BSQ	Day 1	48 (54.5%)	N/A	N/A	N/A
			Day 2	65 (73.9%)	N/A	N/A	N/A
			Question 3: I found the ‘Explanation’ section of the BruxScreen‐Q clear	N/A	3	3	1
			Question 4: I found the ‘Bruxism’ section of the BruxScreen‐Q clear	N/A	3	3	0
			Question 5: I found the ‘Jaw symptoms’ section of the BruxScreen‐Q clear	N/A	3	3	1
	62	BSC	Day 1	37 (58.9%)	N/A	N/A	N/A
			Day 2	47 (75.8%)	N/A	N/A	N/A
			Question 3: I found the ‘Explanation’ section of the BruxScreen‐C clear	N/A	3	3	0.50
			Question 4: I found the ‘Extra‐oral inspection’ section of the BruxScreen‐C clear	N/A	3	3	0.75
			Question 5: I found the ‘Intra‐oral inspection—of the non‐dental tissues’ section of the BruxScreen‐C clear	N/A	3	3	0.56
			Question 6: I found the ‘Intra‐oral inspection—of dental tissues’ section of the BruxScreen‐C clear	N/A	3	3	0.50
Feasibility	88	BSQ	Day 1	49 (55.7%)	N/A	N/A	N/A
			Day 2	64 (72.7%)	N/A	N/A	N/A
			Question 1: I found the BruxScreen‐Q easy to use	N/A	3	3	1
			Question 2: I thought it was an acceptable investment of time to complete the BruxScreen‐Q	N/A	3	3	1
	62	BSC	Day 1	54 (86.3%)	N/A	N/A	N/A
			Day 2	50 (80.6%)	N/A	N/A	N/A
			Question 1: I found the BruxScreen‐C easy to use	N/A	3	3	0.50
			Question 2: I thought it was an acceptable investment of time to complete the BruxScreen‐C	N/A	3	3	0.25

*Note:* The mode, median and interquartile range are displayed for the acceptability and feasibility of the BruxScreen‐C. The outcome was given according to the Likert scale, ranging from zero to four points (viz., strongly disagree [0], disagree [1], neither agree nor disagree [2], agree [3], strongly agree [4]). Cut‐off for the feasibility BruxScreen‐C (a minimum response of ‘agree’ [3 points]); Cut‐off for the acceptability BruxScreen‐C (a minimum response of ‘agree’ [3 points]).

Abbreviations: BSC, BruxScreen‐C; BSQ, BruxScreen‐Q; IQR, interquartile range; Mean (%), mean number of participants; *N*, number of participants; N/A, not applicable.

BruxScreen‐Q: Based on the data from the BruxScreen‐Q, 54.5% of the students (*N* = 48) met the cut‐off for acceptability for Clinical workshop 1. For Clinical workshop 2, 73.9% of the students (*N* = 65) met the cut‐off. The number of participants that met the feasibility cut‐off for Clinical workshop 1 was 55.7% of the students (*N* = 49); for Clinical workshop 2, 72.7% (*N* = 64).

For reading the explanation of the BruxScreen‐Q, the mean duration was 3.9 ± 4.2 min for Clinical workshop 1 and 3.3 ± 3.1 min for Clinical workshop 2. The overall average duration for reading the explanation of the BruxScreen‐Q was 3.6 ± 3.7 min. Furthermore, the average time taken to fill in the BruxScreen‐Q was 5.4 ± 4.2 min for Clinical workshop 1 and 5.1 ± 7.9 min for Clinical workshop 2. The overall average time for filling in the BruxScreen‐Q was 5.3 ± 6.1 min. Additionally, the mean duration of completing all the questionnaires (i.e., BruxScreen‐Q, OBC‐6, TMD‐PS) was 6.1 ± 4.1 min for Clinical workshop 1 and 4.7 ± 2.9 min for Clinical workshop 2. On average, the mean duration to complete all questionnaires was 5.4 ± 3.5 min. These results indicated a decrease in time spent on reading and completing the BruxScreen‐Q.

BruxScreen‐C: Regarding the set cut‐off for the acceptability and feasibility of the BruxScreen‐C, the feasibility scored higher on both days (Clinical workshop 1 and Clinical workshop 2) compared to the acceptability. For the acceptability, an average of 58.9% of the students (*N* = 37) met the cut‐off for Clinical workshop 1, while for Clinical workshop 2, an average of 75.8% of the students (*N* = 47) met the cut‐off. The mean number of participants that met the cut‐off for the feasibility for Clinical workshop 1 was 86.3% (*N* = 54); for Clinical workshop 2, 80.6% (*N* = 50). After Clinical workshop 2, there were still students who scored below the cut‐off for acceptability and feasibility, namely 24.2% of the students (*N* = 15) and 19.4% of the students (*N* = 12), respectively.

The average time spent reading the explanation of the BruxScreen‐C was 2.7 ± 1.9 min during Clinical workshop 1. For Clinical workshop 2, this was 2.2 ± 1.4 min. On average, the mean duration of reading the explanation of the BruxScreen‐C was 2.5 ± 1.5 min. In addition, the mean duration of filling in the BruxScreen‐C was 5.8 ± 2.4 min for Clinical workshop 1. For Clinical workshop 2, this was 3.9 ± 1.8 min. On average, the mean duration of filling in the BruxScreen‐C was 4.8 ± 1.8 min. These findings indicated a decrease in time investment regarding reading and filling in the BruxScreen‐C.

## 4 Discussion

This study aimed to evaluate the reliability, construct validity, acceptability and feasibility of the BruxScreen‐Q and BruxScreen‐C, as well as their concordance in a cohort of Dutch dental students. The findings indicate that the average intra‐rater reliability of the BruxScreen‐Q ranged from fair to substantial. The BruxScreen‐C showed considerable variability for both intra‐ and inter‐rater reliability, ranging from poor reliability to excellent reliability. This variability in clinical assessments may reflect the influence of subjective interpretation, which in turn may be driven by differential salience [20]. The construct validity of the BruxScreen‐Q demonstrated strong alignment with the OBC‐6, except for one item that exhibited a weaker correlation than hypothesised. However, the correlation between the BruxScreen‐Q and the TMD‐PS was weaker than expected for a particular item, possibly due to differences in how these tools assess jaw‐related pain. Acceptability and feasibility results were generally positive, with most students reporting that both BruxScreen‐Q and BruxScreen‐C were easy to use and a reasonable time investment. However, a critical finding of this study is that there was no agreement between BruxScreen‐Q and BruxScreen‐C, reinforcing prior research that suggests self‐reported bruxism and clinical assessments may capture different aspects of the condition. These findings highlight both the strengths and limitations of using self‐reported tools alongside clinical examinations in bruxism assessment.

### 4.1 Reliability

A key limitation of this study was the small sample size per rater for the BruxScreen‐C. Since each student in the dentist role only assessed two participants, this resulted in a sample size of *N* = 2 for intra‐rater reliability. For inter‐rater reliability, two raters independently assessed the same patient, but they only did this for one patient (*N* = 1). Given this design, the reliability may be underestimated due to statistical constraints when using conventional reliability measures such as Cohen's Kappa. To address this issue, the ICC was calculated using GLMMs, which allowed us to model variance across raters and timepoints. Nevertheless, given the current results and the small sample size, the BruxScreen‐C still lacks robust reliability evidence and should be interpreted with caution until further validation is conducted in broader clinical settings.

For future studies, an improved approach is recommended where two experienced dentists will both perform the examinations on all participants and repeat their assessments accordingly over time to establish intra‐ and inter‐rater reliability in the usual manner using a larger and more robust dataset. This approach would provide more conclusive insights into the clinical reliability of the BruxScreen‐C.

### 4.2 Construct Validity

The construct validity analysis between the first question of the BruxScreen‐Q and the OBC‐6 showed positive correlations, with most correlations aligning closely to the a priori formulated hypotheses. Notably, two items of the BruxScreen‐Q demonstrated stronger positive correlations than expected. These findings indicate that these BruxScreen‐Q questions exhibit sufficient validity for assessing bruxism and capturing aspects of bruxism‐related behaviours. Furthermore, the construct validity analysis between the BruxScreen‐Q and the TMD‐PS demonstrated a weak to moderate positive correlation and a medium to large effect size. The correlations and effect sizes were almost all comparable to the a priori hypothesised correlations and effect sizes. However, for two items of the BruxScreen‐Q, the level of correlation was lower than expected. For instance, the correlation between question 2b7 of the BruxScreen‐Q and question 3b of the TMD‐PS was lower than hypothesised (Table 4). While both questions ask about pain in the jaw or temple area, the BruxScreen‐Q focuses on pain frequency during specific activities (i.e., opening the mouth or chewing). In contrast, the TMD‐PS assesses changes in pain resulting from a wide range of jaw movements (i.e., mouth opening or forward or lateral movement) over a specified period. These differences in questioning could explain the discrepancy in the correlation levels.

Three distinct types of validity can be assessed: content validity, criterion validity and construct validity [17]. The BruxScreen was already tested for content validity (i.e., face validity). In this research, the construct validity was analysed. However, criterion validity—which determines whether an instrument accurately predicts an objective gold standard [17, 21]—remains untested for the BruxScreen. In the case of SB, polysomnography (PSG) is the gold standard for diagnosis, but it primarily captures electromyographic activity peaks associated with sleep arousals rather than all jaw muscle activities during the sleep cycle [1]. As a result, validating the BruxScreen against PSG would provide only a partial validation of SB. Future studies should consider exploring alternative biomarker‐based validation approaches to enhance the diagnostic accuracy of the BruxScreen‐C [6].

### 4.3 Acceptability and Feasibility

For this study, the chosen cut‐off values for the acceptability and the feasibility of the BruxScreen‐Q were nine and six points, respectively, while for the BruxScreen‐C, they were twelve and six points. This required participants to select ‘agree’ or higher with each statement to indicate positive usability perceptions. However, it was observed that results were less favourable when using ‘agree’ as the threshold compared to a more neutral cut‐off. Despite this, maintaining the higher threshold provides a more accurate representation of the actual acceptability of the BruxScreen tools in clinical practice.

Interestingly, the response to the acceptability and feasibility items was less positive in the second assessment (Clinical workshop 2), compared to the first assessment (Clinical workshop 1). In the case of the BruxScreen‐C, a possible explanation for this could be that participating in the second BruxScreen workshop was not mandatory for the students' education as per the ethical clearance, although students were present due to a subsequent mandatory workshop. The lack of intrinsic motivation may have led to lower engagement and less favourable responses during Clinical workshop 2. Previous research suggests that forced participation in academic settings can result in lower enthusiasm and less accurate reflections of true usability perceptions [22].

### 4.4 Concordance Between the BruxScreen‐Q and BruxScreen‐C

To examine the concordance between the BruxScreen‐Q and the BruxScreen‐C, only complete data sets from both workshops (Clinical workshop 1 and Clinical workshop 2) were included in the analysis. However, a notable inconsistency was observed in the BruxScreen‐Q responses between the two workshops, suggesting response bias due to students becoming more familiar with the study design after the first session (Clinical workshop 1). Awareness of the study procedures may have led participants to modify their self‐reported responses, affecting the reliability of the comparison between the BruxScreen‐Q and the BruxScreen‐C [23]. This is an important limitation, as in real‐world clinical settings, patients typically complete the BruxScreen‐Q only once.

In the current study, only 5.6% of the participating students (*N* = 3) present themselves with bruxism based on both self‐report and clinical examination. Based on the data of this study, there is no agreement between the BruxScreen‐Q and the BruxScreen‐C. This could be explained as follows. Various studies have found discrepancies between self‐report and clinical assessment of bruxism. For example, it was found that different approaches to measure bruxism (viz., self‐report and device‐based approaches) lead to different outcomes [24]. In another study [10], it was tested if there was a correlation between self‐reported bruxism on the one hand, and the combination of a clinical examination with a questionnaire on the other hand. Based on that study, for diagnosing AB (clenching type), there was a strong positive correlation between self‐reported bruxism and clinically based bruxism. However, there was a very low agreement for AB (grinding type). Furthermore, there were lower levels of agreement for diagnosing grinding or clenching while sleeping. It was hypothesised that self‐reported bruxism measures a different construct of bruxism than the clinical measurements, whereby the self‐reported measurements also reflect patients' psychological state and self‐beliefs of bruxism [24]. Therefore, the BruxScreen‐Q and the BruxScreen‐C may measure different aspects of bruxism and should be considered complementary rather than interchangeable tools.

### 4.5 Limitations

The study has several design limitations that should be considered when interpreting the results. First, the participants consist solely of dental students, limiting the external validity and the generalizability of the results to broader populations [25]. To address this limitation, future studies should evaluate the BruxScreen in a more diverse participant group and include assessments conducted by experienced dentists or other healthcare professionals to determine the reliability and applicability across different clinical settings.

Second, the cut‐off points for both subject‐based bruxism and clinically based bruxism were pre‐defined based on the clinical experience and consensus within the research team. However, for future studies, Receiver Operating Characteristic (ROC) curve analysis is recommended to determine the most appropriate cut‐off points [26].

## 5 Conclusion

The BruxScreen demonstrates acceptable reliability, construct validity, acceptability and feasibility in assessing both self‐reported bruxism and clinically measured bruxism in a student population. However, there is a discrepancy between subject‐based bruxism and clinically based bruxism, suggesting that self‐report and clinical examination may not fully align. Overall, the current findings mainly support the screening potential of the BruxScreen, while its diagnostic accuracy remains to be established.

## Author Contributions

L.J.K., M.C.V. and F.L. contributed to the conception of this project. L.J.K. drafted the manuscript. All authors critically revised the manuscript and approved the final version. They are all responsible for all aspects of the work.

## Conflicts of Interest

The authors declare no conflicts of interest.

## Data Availability

The data that support the findings of this study are available on request from the corresponding author. The data are not publicly available due to privacy or ethical restrictions.

## Appendix A

### Acceptability and Feasibility BruxScreen‐Q

Please indicate to what extent you agree with the statements:

1. I found the BruxScreen‐Q easy to use
  ○ Strongly disagree (0 pt)
  ○ Disagree (1 pts)
  ○ Neutral (2 pts)
  ○ Agree (3 pts)
  ○ Strongly agree (4 pts)
2. I thought it was an acceptable investment of time to complete the BruxScreen‐Q
  ○ Strongly disagree (0 pt)
  ○ Disagree (1 pts)
  ○ Neutral (2 pts)
  ○ Agree (3 pts)
  ○ Strongly agree (4 pts)
3. I found the ‘Explanation’ section of the BruxScreen‐Q clear
  ○ Strongly disagree (0 pt)
  ○ Disagree (1 pts)
  ○ Neutral (2 pts)
  ○ Agree (3 pts)
  ○ Strongly agree (4 pts)
4. I found the ‘Bruxism’ section of the BruxScreen‐Q clear
  ○ Strongly disagree (0 pt)
  ○ Disagree (1 pts)
  ○ Neutral (2 pts)
  ○ Agree (3 pts)
  ○ Strongly agree (4 pts)
5. I found the ‘Jaw symptoms’ section of the BruxScreen‐Q clear
  ○ Strongly disagree (0 pt)
  ○ Disagree (1 pts)
  ○ Neutral (2 pts)
  ○ Agree (3 pts)
  ○ Strongly agree (4 pts)

How long did it take to read the explanation of the BruxScreen‐Q? (XX.X min)

How long did it take to complete the BruxScreen‐Q? (XX.X min)

How long did it take to complete the BruxScreen‐Q, OBC‐6 and TMD‐PS? (XX.X min)

Do you have any comments or suggestions? (Open field)

### Acceptability and Feasibility BruxScreen‐C

Please indicate to what extent you agree with the statements:

1. I found the BruxScreen‐C easy to use
  ○ Strongly disagree (0 pt)
  ○ Disagree (1 pts)
  ○ Neutral (2 pts)
  ○ Agree (3 pts)
  ○ Strongly agree (4 pts)
2. I thought it was an acceptable investment of time to complete the BruxScreen‐C
  ○ Strongly disagree (0 pt)
  ○ Disagree (1 pts)
  ○ Neutral (2 pts)
  ○ Agree (3 pts)
  ○ Strongly agree (4 pts)
3. I found the ‘Explanation’ section of the BruxScreen‐C clear
  ○ Strongly disagree (0 pt)
  ○ Disagree (1 pts)
  ○ Neutral (2 pts)
  ○ Agree (3 pts)
  ○ Strongly agree (4 pts)
4. I found the ‘Extra‐oral inspection’ section of the BruxScreen‐C clear
  ○ Strongly disagree (0 pt)
  ○ Disagree (1 pts)
  ○ Neutral (2 pts)
  ○ Agree (3 pts)
  ○ Strongly agree (4 pts)
5. I found the ‘Intra‐oral inspection—of the non‐dental tissues’ section of the BruxScreen‐C clear
  ○ Strongly disagree (0 pt)
  ○ Disagree (1 pts)
  ○ Neutral (2 pts)
  ○ Agree (3 pts)
  ○ Strongly agree (4 pts)
6. I found the ‘Intra‐oral inspection—of dental tissues’ section of the BruxScreen‐C clear
  ○ Strongly disagree (0 pt)
  ○ Disagree (1 pts)
  ○ Neutral (2 pts)
  ○ Agree (3 pts)
  ○ Strongly agree (4 pts)

How long did it take to read the explanation of the BruxScreen‐C? (XX.X min)

How long did it take to complete the BruxScreen‐C? (XX.X min)

Do you have any comments or suggestions? (Open field)

## Appendix B

**TABLE B1:** Hypotheses for construct validity analysis.

Bruxscreen‐Q question	Comparable validated question		Statistical test	Hypothesis
1a. How often do you clench your teeth during sleep?	OBC‐6	1. How often do you clench or grind teeth when asleep, based on any information you may have?	Spearman's rank correlation	0.4 (moderate)
1b. How often do you grind your teeth during sleep?	OBC‐6	1. How often do you clench or grind teeth when asleep, based on any information you may have?	Spearman's rank correlation	0.4 (moderate)
1c. How often do you clench your teeth while awake?	OBC‐6	4. How often do you clench teeth together during waking hours?	Spearman's rank correlation	0.6 (strong)
1d. How often do you grind your teeth while awake?	OBC‐6	3. How often do you grind your teeth during waking hours?	Spearman's rank correlation	0.6 (strong)
1e. How often do you light press, touch or hold teeth together while awake other than while eating (i.e., contact between upper and lower teeth)?	OBC‐6	5. How often do you press, touch or hold teeth together other than while eating (i.e., contact between upper and lower teeth)	Spearman's rank correlation	0.6 (strong)
2a. How often do you experience pain in your temple, face or jaw point upon awaking?	TMD‐PS	2. In the last 30 days, have you had pain or stiffness in your jaw on awakening?	Mann–Whitney *U* test	0.4 (moderate)
2a. How often do you experience pain in your temple, face or jaw point upon awaking at any other time?	TMD‐PS	1. In the last 30 days, which of the following describes any pain in your jaw or temple area on either side? (no pain, pain comes and goes, pain is always present)	Mann–Whitney *U* test	0.4 (moderate)
2b. How often do you experience pain in your temple, face or jaw point when you open your mouth or chew? (Answers meals related)	TMD‐PS	3a. In the last 30 days, did chewing hard or tough food change any pain (i.e., make it better or make it worse) in your jaw or temple area on either side?	Spearman's rank correlation	0.4 (moderate)
2b. How often do you experience pain in your temple, face or jaw point when you open your mouth or chew? (Answers related to opening the mouth)	TMD‐PS	3b. In the last 30 days, did opening your mouth or moving your jaw forward or to the side change any pain (i.e., make it better or make it worse) in your jaw or temple area on either side?	Spearman's rank correlation	0.4 (moderate)

*Note:* Statistical tests and hypotheses for the construct validity analysis. The hypotheses were based on our clinical and research experience.

Abbreviations: OBC‐6, Oral Behaviour Checklist‐6; TMD‐PS, Temporomandibular Disorder Pain Screener.

## References

[1] F. Lobbezoo , J. Ahlberg , A. G. Glaros , et al., “Bruxism Defined and Graded: An International Consensus,” Journal of Oral Rehabilitation 40, no. 1 (2013): 2–4, 10.1111/joor.12011. 23121262

[2] F. Lobbezoo , J. Ahlberg , K. G. Raphael , et al., “International Consensus on the Assessment of Bruxism: Report of a Work in Progress,” Journal of Oral Rehabilitation 45, no. 11 (2018): 837–844, 10.1111/joor.12663. 29926505 PMC6287494

[3] P. Wetselaar , E. Vermaire , F. Lobbezoo , and A. A. Schuller , “The Prevalence of Awake Bruxism and Sleep Bruxism in the Dutch Adult Population,” Journal of Oral Rehabilitation 46, no. 7 (2019): 617–623, 10.1111/joor.12787. 30830687 PMC6849828

[4] D. Manfredini , J. Ahlberg , P. Wetselaar , P. Svensson , and F. Lobbezoo , “The Bruxism Construct: From Cut‐Off Points to a Continuum Spectrum,” Journal of Oral Rehabilitation 46, no. 11 (2019): 991–997, 10.1111/joor.12833. 31264730

[5] M. C. Verhoeff , F. Lobbezoo , J. Ahlberg , et al., “Updating the Bruxism Definitions: Report of an International Consensus Meeting,” Journal of Oral Rehabilitation 52 (2025): 1335–1342, 10.1111/joor.13985. 40312776 PMC12408978

[6] D. Manfredini , J. Ahlberg , G. Aarab , et al., “Towards a Standardized Tool for the Assessment of Bruxism (STAB)—Overview and General Remarks of a Multidimensional Bruxism Evaluation System,” Journal of Oral Rehabilitation 47, no. 5 (2020): 549–556, 10.1111/joor.12938. 31999846

[7] D. Manfredini , J. Ahlberg , G. Aarab , et al., “Standardised Tool for the Assessment of Bruxism,” Journal of Oral Rehabilitation 51, no. 1 (2024): 29–58, 10.1111/joor.13411. 36597658

[8] F. Lobbezoo , J. Ahlberg , M. C. Verhoeff , A. Bracci , L. Nykänen , and D. Manfredini , “Translation and Cultural Adaptation of the Standardized Tool for the Assessment of Bruxism (STAB) and the Bruxism Screener (BruxScreen): A 12‐Step Guideline,” Journal of Oral Rehabilitation 51, no. 1 (2024): 67–73, 10.1111/joor.13602. 37749858

[9] F. Lobbezoo , J. Ahlberg , M. C. Verhoeff , et al., “The Bruxism Screener (BruxScreen): Development, Pilot Testing and Face Validity,” Journal of Oral Rehabilitation 51, no. 1 (2024): 59–66, 10.1111/joor.13442. 36843424

[10] D. A. Paesani , F. Lobbezoo , C. Gelos , L. Guarda‐Nardini , J. Ahlberg , and D. Manfredini , “Correlation Between Self‐Reported and Clinically Based Diagnoses of Bruxism in Temporomandibular Disorders Patients,” Journal of Oral Rehabilitation 40, no. 11 (2013): 803–809, 10.1111/joor.12101. 24112029

[11] E. L. Schiffman , R. Ohrbach , E. Truelove , et al., “Diagnostic Criteria for Temporomandibular Disorders (DC/TMD) for Clinical and Research Applications: Recommendations of the International RDC/TMD Consortium Network* and Orofacial Pain Special Interest Group†,” Journal of Oral & Facial Pain and Headache 28, no. 1 (2014): 6–27, 10.11607/jop.1151. 24482784 PMC4478082

[12] R. Ohrbach , “The Oral Behavior Checklist,” (2013), accessed November 19, 2024, https://inform‐iadr.com/wp‐content/uploads/2024/01/Oral‐Behavior‐Checklist_2013‐05‐12.pdf.

[13] P. Wetselaar and F. Lobbezoo , “The Tooth Wear Evaluation System: A Modular Clinical Guideline for the Diagnosis and Management Planning of Worn Dentitions,” Journal of Oral Rehabilitation 43, no. 1 (2016): 69–80, 10.1111/joor.12340. 26333037

[14] C. Scheel , J. Mecham , V. Zuccarello , and R. Mattes , “An Evaluation of the Inter‐Rater and Intra‐Rater Reliability of Occupro's Functional Capacity Evaluation,” Work 60, no. 3 (2018): 465–473, 10.3233/wor-182754. 30040785 PMC6087436

[15] M. L. McHugh , “Interrater Reliability: The Kappa Statistic,” Biochemia Medica 22 (2012): 276–282, 10.11613/bm.2012.031. 23092060 PMC3900052

[16] T. K. Koo and M. Y. Li , “A Guideline of Selecting and Reporting Intraclass Correlation Coefficients for Reliability Research,” Journal of Chiropractic Medicine 15, no. 2 (2016): 155–163, 10.1016/j.jcm.2016.02.012. 27330520 PMC4913118

[17] L. B. Mokkink , C. B. Terwee , D. L. Patrick , et al., “The COSMIN Study Reached International Consensus on Taxonomy, Terminology, and Definitions of Measurement Properties for Health‐Related Patient‐Reported Outcomes,” Journal of Clinical Epidemiology 63, no. 7 (2010): 737–745, 10.1016/j.jclinepi.2010.02.006. 20494804

[18] S. N. Papageorgiou , “On Correlation Coefficients and Their Interpretation,” Journal of Orthodontics 49, no. 3 (2022): 359–361, 10.1177/14653125221076142. 36017900 PMC9420886

[19] Mann‐Whitney U‐Test , “DATAtab: Online Statistics Calculator,” accessed October 29, 2024, https://datatab.net/tutorial/mann‐whitney‐u‐test.

[20] G. Gauthier , C. St‐Onge , and W. Tavares , “Rater Cognition: Review and Integration of Research Findings,” Medical Education 50, no. 5 (2016): 511–522, 10.1111/medu.12973. 27072440

[21] K. Nikolopoulou , What Is Criterion Validity? | Definition & Examples (Scribbr, 2023), https://www.scribbr.com/methodology/criterion‐validity/.

[22] S. Saeed and D. Zyngier , “How Motivation Influences Student Engagement: A Qualitative Case Study,” Journal of Education and Learning 1, no. 2 (2012): 252–267, 10.5539/jel.v1n2p252.

[23] P. Bhandari , Demand Characteristics | Definition, Examples, & Control (Scribbr, 2023), https://www.scribbr.com/research‐bias/demand‐characteristics/.

[24] T. Chattrattrai , Sleep Bruxism: Associations and Comorbid Conditions. Thesis. (Universiteit van Amsterdam, 2024).

[25] C. L. Ramspek , K. J. Jager , F. W. Dekker , C. Zoccali , and M. Van Diepen , “External Validation of Prognostic Models: What, Why, How, When and Where?,” Clinical Kidney Journal 14, no. 1 (2020): 49–58, 10.1093/ckj/sfaa188. 33564405 PMC7857818

[26] K. Hajian‐Tilaki , “Receiver Operating Characteristic (ROC) Curve Analysis for Medical Diagnostic Test Evaluation,” Caspian Journal of Internal Medicine 4, no. 2 (2013): 627–635. 24009950 PMC3755824

